# Analytical study of fractional solitons in three dimensional nonlinear evolution equation within fluid environments

**DOI:** 10.1038/s41598-025-12576-5

**Published:** 2025-10-10

**Authors:** M. Elsaid Ramadan, Hamdy M. Ahmed, Abeer S. Khalifa, Karim K. Ahmed

**Affiliations:** 1 Department of Mathematics, Faculty of Science, Islamic University of Madinah, Medina, Saudi Arabia; 2 Department of Physics and Engineering Mathematics, Higher Institute of Engineering, El Shorouk Academy, Cairo, P.O. Box 11837, Egypt; 3 Department of Mathematics, Faculty of Basic Sciences, The German University in Cairo (GUC), Cairo, Egypt; 4 Department of Mathematics, Faculty of Engineering, German International University (GIU), New Administrative Capital Cairo, Egypt

**Keywords:** Conformable fractional derivative, Optical solitons, The nonlinear (3+1)-dimensional evolution equation, NLPDEs, Modified extended mapping method, Materials for optics, Engineering, Optics and photonics, Physics, Mathematics and computing, Applied mathematics

## Abstract

This study investigates a nonlinear (3+1)-dimensional evolution equation in the conformable fractional derivative (CFD) sense, which may be useful for comprehending how waves change in water bodies like seas and oceans. Certain intriguing non-linear molecular waves are linked to solitons and other modified waves that result from the velocity resonance condition. The characteristic lines of each wave component show that these waves have a set spacing throughout their propagation. We start by using the proposed model and the modified extended mapping technique. We also conduct an analysis of the various solutions, including bright, dark, and singular solitons; periodic wave solutions; exponential wave solutions; hyperbolic solutions; Jacobi elliptic function (JEF) solutions; Weierstrass elliptic doubly periodic solutions; and rational wave solutions. By clarifying how fractional-order dynamics modulate wave amplitude and dispersion features, the resulting solutions allow for a more realistic depiction of complicated fluid behaviors seen in empirical investigations of coastal and stratified oceanic settings. To provide them with a physical comprehension of the obtained solutions, some of the extracted solutions are illustrated visually. The obtained solutions reveal how fractional-order effects influence wave stability, energy transport, and interaction dynamics in fluid systems, offering practical insights for modeling coastal processes, pollutant dispersion, and wave-current interactions in real marine environments.

## Introduction

The potential uses of solitons in high-speed communication systems have drawn a lot of interest in their study in optical fibers. Since they can retain their amplitude and shape while propagating, they are perfect for delivering information with reliability. Solitons have been studied by several researchers; for instance, the authors in^1^ investigated a bright, solitary soliton in a nonlinear Schrödinger equation with spatio-temporal dispersions in (2+1) dimensions. The significance of ions in optical systems with dispersive effects was the main focus of the authors’ study of ion formation, properties, and behavior. In reference^2^, solitons derived from the Schrödinger equation’s nonlinearity were described. The study’s identification of novel types of solitons in this system aided in understanding their characteristics and behavior. In^3^, the authors created the new solitons for the higher-order formula known as Sasa-Satsuma. The authors discussed the soliton dynamics of the Vakhneno equation in an optical fiber system in^4^. In^5^, the authors talked about the Maccari system’s bifurcation and soliton resonance. For the Lakshmanan-Daniel system, the authors in^6^ determined the soliton resonance. There is still a lot of interest in discovering new solitons, comprehending their mechanisms, and discovering their optical transmission properties and stability. This is understandable given the wide range of real-world uses for these localized structures in nonlinear engineering and research^7–12^. Nonlinear wave types are naturally occurring phenomena that merit particular consideration as solutions to nonlinear evolution equations (NLEEs), taking into account the solitons^13–15^. Many domains depend on these waves, such as nonlinear optics^16,17^, plasma^18^, and hydrodynamics^19^. NLEE’s solutions can be obtained mathematically using various techniques, for example, the modified extended direct algebraic technique^20^, the improved modified extended tanh function technique^21^, the extended F-expansion technique^22^, the modified extended mapping technique^23^, and others. Many of these techniques are utilized extensively for a variety of high-dimensional non-linear evolution equations^24–27^. Recently, certain high-dimensional models have revealed modified waves with time-varying characteristics^28,29^. During propagation, these waves exhibit intriguing shape-changing properties. It should be mentioned that wave collapses and high-dimensional modified waves have comparable time-varying characteristics^30^. Akinyemi has recently suggested a unique(2+1)-dimensional NLEE that was suggested in^31^. This equation was expanded to a new (3+1)-dimensional form by Wazwaz et al., based on its physical relevance, which he dimensioned in^32^. Many researchers studied this new extended (3+1)-dimensional famous equation of Wazwaz in several areas of science, such as^33^, which provided an examination of many hybrid molecules, breathers, altered molecular waves, and solitons. Dispersion is one of the main elements influencing how solitons behave in optical fibers. Dispersion is the process by which distinct wavelengths propagate at varying speeds, causing the pulse to gradually expand or shrink. Dispersion is still a major problem in optical fiber system design and optimization, even though it may be somewhat controlled with the use of dispersion compensation techniques^34^. The investigation of extremely dispersive gap solitons has gained attention in recent years. Localized solutions known as gap solitons are found within optical fibers that contain periodic or quasi-periodic structures like fiber Bragg gratings or photonic crystals. In the linear dispersion relation’s spectral gaps, when the propagation of linear waves is prohibited, these solitons can arise. Solitons are the result of the interplay between the dispersive reflectivity of the periodic structure and the optical fiber’s nonlinearity^35–37^.

The use of fractional calculus to solve nonlinear evolution equations has garnered a lot of interest lately because of its potential to explain memory and genetic characteristics in intricate physical systems. Introduced by Khalil et al.^38^, the conformable fractional derivative extends the differentiation order to non-integer values while maintaining a number of integer-order calculus properties, such as the product, quotient, and chain rules. This makes it easier to apply the derivative to nonlinear differential equations. The fractional-order model physically captures the anomalous diffusion, hereditary memory, and progressive relaxation phenomena that are frequently seen in complicated fluid systems^39^. These effects result from long-range correlations and internal microstructures in the fluid, which are not well captured by conventional models.

Mathematicians, engineers, and physicists are interested in the use of fractional-order differential equations (FODEs) in most research nowadays, which is managed by a variety of approaches^40–43^. The foregoing makes it evident how those realistic models might uncover fresh occurrences in such systems that haven’t been found before. Some accurate NLEE solutions, fractional nonlinear differential equations (FNDEs) in particular, have lately attracted increased attention. For a more thorough understanding of the physical mechanisms behind intricate nonlinear occurrences and for their ongoing use in physical issues that arise in real-time, the solution of a fractional differential equation is essential. As a result, analytical and numerical solutions to conformable equations have gained popularity. The dynamics of the solitons and fractional derivatives are a more accurate way to depict long-range interactions. The CFD features on the third-order Kdv equation and the telegraph equation were covered by the authors in^44^. In order to investigate the dynamics of solitons, the conformable derivative was also covered in^45^ on the evolution equation in optical fiber systems. The CFD of the modified Benjamin-Mahony equation was covered by the authors in^46^. The CF coupled type Boussinesq-Burger equation was covered in the publication^47^. Numerous additional types of fractional derivatives, including beta derivatives^48,49^, have been examined to determine how fractional order affects solitons.

A more faithful representation of the intricate rheological behavior frequently seen in actual fluid media is made possible by the conformable fractional derivative framework, which incorporates memory effects and nonlocal interactions into the governing equations of fluid flow. The complicated behavior of the fluid medium under study is captured in this work by using the conformable fractional derivative. The addition of memory effects and non-local interactions, which are essential for correctly characterizing several physical processes in viscoelastic and non-Newtonian fluids, is made possible by fractional derivatives, in contrast to classical derivatives^50,51^.

Solitons, multi-solitons, and bifurcation solutions for fractional models occurring in fluid dynamics and nonlinear optics have been effectively derived analytically in a number of research studies. Akram et al.^52^ examined stability characteristics of dispersive optical solitons within the framework of the fractional Schrödinger–Hirota equation, while Ahmad et al.^53^ examined resonant multi-soliton solutions of time-fractional coupled nonlinear Schrödinger equations in optical fibers. New solutions to the conformable fractional perturbed Gerdjikov-Ivanov equation were derived by Zulfiqar et al.^54^, exposing rich optical features under fractional perturbations. The interaction between bifurcation, chaos, and soliton solutions in fractional Hirota models was also studied by Javed et al.^55^, while Ahmad and Mohyud-Din^56^ created effective methods to address highly nonlinear fractional PDEs. Inspired by these developments, the research is expanded to fractional models in fluid media in this work, emphasizing the physical interpretation of wave dynamics under fractional-order effects and their applicability to actual fluid systems.

According to this study, we suggest the handling of (3+1)-dimensional NLEE Eq.(1)^33^, which represents a generalized model of a physical that depends on both spatial and temporal variables, such as wave propagation, heat conduction, or fluid flow with higher-order and nonlinear effects, possibly in a heterogeneous or anisotropic medium. We utilize the modified extended mapping method^11^ but with the introduction of a conformable fraction term to the NLEE Eq.(1) through the model transformation in the applied method. Using this technique could represent generalized diffusion or wave propagation in a multidimensional medium. Also, one can investigate the nonlinear wave phenomena and enable the obtaining of a variety of solitons and other precise solutions. This combination is considered a system that is previously unused and offers a superior construction of several solitary wave solution types, as well as others like Jacobi elliptic function solutions, Weierstrass function solutions, hyperbolic solutions, exponential wave solutions, bright, dark, singular solitons, periodic wave solutions, and rational wave solutions. The extracted solutions attest to the potency and effectiveness of the current method. To further illustrate, 3D and 2D simulations are shown along with the nature of the solutions that were discovered.

1 $$\begin{aligned} \begin{aligned} D^\alpha _t \Phi _x+\delta _1 \Phi _{xx}+\delta _2 (\Phi ^2)_{xx}+\delta _3 \Phi _{xxxx}+\delta _4 \Phi _{yy}+\sigma \Phi _{xy}+\beta \Phi _{xz}+\gamma \Phi _{yz}+\rho \Phi _{zz}=0, \end{aligned} \end{aligned}$$

where $\delta _1,\ \delta _2,\ \delta _3,\ \delta _4,\ \sigma ,\ \beta ,\ \gamma$ and $\rho$ act as real constants, *t* is the time variable, $\Phi (x, y, z, t)$ is a total real function of these coordinates, and *x*, *y*, *z* are spatial coordinates. Several physical factors that are frequently seen in complex media are incorporated into the examined (3+1)-dimensional nonlinear fractional evolution equation. Nonlocal memory effects and anomalous diffusion or dispersion processes, which are common in many real-world systems such as viscoelastic fluids, plasma, and porous media, are reflected in the inclusion of the conformable fractional derivative $D^\alpha _t$ in time. The higher-order dispersion term $\Phi _{xxxx}$ captures substantial dispersive corrections necessary for modeling broadband wave packets, while the term $(\Phi ^2)_{xx}$ denotes quadratic nonlinearity, which controls nonlinear self-interaction and energy exchange processes. Anisotropic dispersion and multidirectional coupling of wave modes are taken into account by the mixed spatial derivative terms $\Phi _{xy},\ \Phi _{xz},\ \Phi _{yz}$ and the second-order dispersion terms $\Phi _{xx},\ \Phi _{yy},\ \Phi _{zz}$, which enable the model to replicate complex interactions in multi-dimensional physical systems. This kind of equation may be used to explain nonlinear wave propagation in fluids, acoustic waves in elastic media, plasma waves, Bose-Einstein condensates, and nonlinear optics, where wave dynamics are greatly influenced by both spatial anisotropy and memory-dependent development.

Furthermore, we define

$$\begin{aligned} D^\alpha _t \Phi (x,y,z,t)=\lim _{{\delta \rightarrow 0}} \frac{{\Phi (x,y,z,t + \delta t^{1-\alpha }) - \Phi (x,y,z,t)}}{\delta },~~ t>0, \end{aligned}$$

which is a CFD with order $0< \alpha \le 1$.

### Contribution and novelty of this study

This work’s primary contribution is the delivery of novel, precise solutions that explain the intricate dynamics of multidimensional nonlinear waves, including soliton structures and their stability characteristics. With applications in oceanography (to simulate surface and internal waves), nonlinear optics (for pulse propagation in optical fibers), plasma physics (for wave interactions in plasma media), and even seismology and biomedical engineering (where wave phenomena are crucial), these findings advance our theoretical understanding of nonlinear wave propagation.

As opposed to other studies, this study employs this method to model generalized wave propagation and diffusion in multidimensional media as opposed to lower-dimensional models or limited classes of solutions that were considered in other works. As compared to other approaches, this scheme permits a broader class of nonlinear wave phenomena to be considered and several exact solutions to be derived. These are various types of solitons (bright, dark, solitary), Jacobi elliptic function solution, Weierstrass function solution, hyperbolic and exponential wave forms, periodic structures, and rational solutions. The results presented demonstrate greater capability and flexibility of the method proposed as compared to other published methods.

Besides, through systematically transforming the original equations into solvable ones without making strong restrictions on the parameters, the modified extended mapping method is more flexible in handling complex nonlinearities and fractional orders than other approaches like Hirota’s bilinear method or exp-function method extensively used in^57,58^. Therefore, physically more meaningful solutions are available to explain a wider range of physical phenomena, including ion-acoustic waves in plasma^58^, solitary structures in DNA modeling^57^, interaction patterns in Boussinesq-type systems^59^, and wave propagation in Bose-Einstein condensates^60,61^. Along the same lines, the solutions thus obtained demonstrate modified extended mapping method’s broader application and strength compared to various standard techniques.

This article is organized into several sections. An overview of the suggested model and its theoretical underpinnings is given in Section 1. Section 2 outlines the main concepts of the modified extended mapping approach. In Section 3, all findings are summarized using the Wolfram Mathematica software, which does all the symbolic computations. In Section 4, multiple patterns of dynamic waves in various solitons are represented graphically using 3-D and 2-D simulations. The work conclusions are finally reported in Section 5.

## The modified extended mapping technique summary

The modified extended mapping technique is introduced in this section^11^. Taking into account the subsequent NLPDE:

2 $$\begin{aligned} \mathbb {Q} \left( \Phi , D^\alpha _t \Phi , \Phi _{x}, \Phi _{y}, \Phi _{z},\Phi _{xx}, \Phi _{tt}, D^\alpha _t \Phi _x, \Phi _{xy}, ...\right) = 0, \end{aligned}$$

where $\mathbb {Q}$ represents the equivalent partial derivatives for space and time of the polynomial function $\Phi (x,y,z,t)$.

**Step-1:** The transformation of the traveling wave is applied as follows:

3 $$\begin{aligned} \Phi (x,y,z,t)= \varUpsilon (\xi ), \qquad \xi =a\ x+b\ y+c\ z-\kappa \ \frac{ t^{\alpha }}{\alpha },\ \ \end{aligned}$$

where arbitrary constants *a*, *b*, and *c* are used. While $\frac{ t^{\alpha }}{\alpha }$ shows the fractional term in the transformation meaning. The wave speed that will be measured later is represented by the real constant $\kappa$.

Eq.(3) can be substituted into Eq.(2) to convert it into the non-linear ordinary differential equation (NLODE) that is mentioned below:

4 $$\begin{aligned} \mathbb {T} (\varUpsilon , \varUpsilon ^\prime , \varUpsilon ^{\prime \prime },\varUpsilon ^{\prime \prime \prime } \ldots ) = 0. \end{aligned}$$

**Step-2:** Eq. (4) then has a solution that can be expressed as:

5 $$\begin{aligned} \varUpsilon (\xi )=\sum _{i=0}^{\mathbb {N}}{\mathbb {A}_i \mathbb {W}^i(\xi )}+\sum _{i=-1}^{-\mathbb {N}}{\mathbb {B}_{-i} \mathbb {W}^i(\xi )}+\sum _{i=2}^{\mathbb {N}}{\mathbb {C}_i \mathbb {W}^{i-2}(\xi )\mathbb {W}'(\xi )}+\sum _{i=-1}^{-\mathbb {N}}{\mathbb {D}_{-i} \mathbb {W}^{i}(\xi )\mathbb {W}'(\xi )}, \end{aligned}$$

where $\mathbb {W}(\xi )$ meets the following auxiliary equation condition and $\mathbb {A}_i,\mathbb {B}_{-i},\mathbb {C}_i,\mathbb {D}_{-i}$ are real constants to be estimated:

6 $$\begin{aligned} \mathbb {W}'(\xi )=\sqrt{\tau _0+\tau _1 \mathbb {W}(\xi )+\tau _2 \mathbb {W}^2(\xi )+\tau _3 \mathbb {W}^3(\xi )+\tau _4 \mathbb {W}^4(\xi )+\tau _6 \mathbb {W}^6(\xi )}, \end{aligned}$$

that contains constants $\tau _j,\ (j = 0, 1, 2, 3, 4, 6)$.

**Step-3:** The positive number $\mathbb {N}$ can be found using Eq. (4) and the concept of balance between the highest-order derivatives and the highest-order nonlinear terms.

**Step-4:** The purported solution from Eqs. (5) and (6) is inserted in Eq. (4), and the coefficients of $\mathbb {W}'^i(\xi )\mathbb {W}^j(\xi )$ ($i=0,1$; $j=0,\pm 1,\pm 2,...$) are then equalized to zero, $\mathbb {A}_i,\mathbb {B}_{-i},\mathbb {C}_i,\mathbb {D}_{-i} ~\text {and}~\kappa$ are then considered a group of nonlinear algebraic equations that can be solved using Maple or Mathematica computing programs. In order to get several precise solutions to Eq. (2), we must first determine the unknown constants $\mathbb {A}_i,\mathbb {B}_{-i},\mathbb {C}_i~\text {and}~\mathbb {D}_{-i}$.

## Novel of soliton solutions and some other solutions

The solutions of the wave transformation in (3) is assumed to (1), considering $\kappa$ and $\alpha \ne 0$, where $\varUpsilon (\xi )$ acts as the amplitude term of the solution. Substituting by Eqs.(3) into Eq.(1), yields the following:

7 $$\begin{aligned} \begin{aligned} \left( a b \sigma +a c\beta +b c \gamma -a \kappa +a^2 \delta _1+b^2 \delta _4+c^2 \rho \right) \varUpsilon ''+2 a^2 \delta _2 \left( \left( \varUpsilon '\right) ^2+\varUpsilon \varUpsilon ''\right) + a^4 \delta _3 \varUpsilon ^{(4)}=0. \end{aligned} \end{aligned}$$

The following outcome can be obtained by taking the zero integration constant and integrating Eq.(7) twice:

8 $$\begin{aligned} \begin{aligned} \left( a b \sigma +a c\beta +b c \gamma -a \kappa +a^2 \delta _1+b^2 \delta _4+c^2 \rho \right) \varUpsilon +a^2 \delta _2 \varUpsilon ^2 +a^4 \delta _3 \varUpsilon ''=0. \end{aligned} \end{aligned}$$

Thus, by applying the homogeneous balancing principle outlined in section 2 to $\varUpsilon ''$ and $\varUpsilon ^2$, we obtain $\mathbb {N}=2$ and the general solution from Eq. (5) as follows:

9 $$\begin{aligned} \begin{aligned} \varUpsilon (\xi )=\mathbb {A}_0+\mathbb {A}_1 \mathbb {W}(\xi )+\mathbb {A}_2 \mathbb {W}(\xi )^2+\frac{\mathbb {B}_1}{\mathbb {W}(\xi )}+\frac{\mathbb {B}_2}{\mathbb {W}(\xi )^2}+\mathbb {C}_2 \mathbb {W}'(\xi )+\frac{\mathbb {D}_1 \mathbb {W}'(\xi )}{\mathbb {W}(\xi )}+\frac{\mathbb {D}_2 \mathbb {W}'(\xi )}{\mathbb {W}(\xi )^2}, \end{aligned} \end{aligned}$$

using Eq. (9) with the aid of the auxiliary (6), by inserting them Eq. (8), the following results can be obtained by using Mathematica to resolve a set of non-linear algebraic equations generated when coefficients of identical powers are consolidated and equated to zero:

**Case (1):** When $\tau _0 =\tau _1 = \tau _3 =\tau _6 = 0$, the subsequent solution sets were found:

(**1.1**): $$\mathbb {A}_0= \mathbb {A}_1=\mathbb {B}_1=\mathbb {B}_2=\mathbb {C}_2=\mathbb {D}_1=\mathbb {D}_2=0,\ \mathbb {A}_2= \frac{3 \tau _4 \left( a b \sigma +a c\beta +b c \gamma -a \kappa +a^2 \delta _1+b^2 \delta _4+c^2 \rho \right) }{2 a^2 \delta _2 \tau _2},$$
  $$\delta _3= -\frac{\left( a b \sigma +a c\beta +b c \gamma -a \kappa +a^2 \delta _1+b^2 \delta _4+c^2 \rho \right) }{4 a^4 \tau _2}.$$
(**1.2**): $$\mathbb {A}_0= \mathbb {A}_1=\mathbb {B}_1=\mathbb {B}_2=\mathbb {D}_1=\mathbb {D}_2=0,\ \mathbb {A}_2= \frac{3 \tau _4 \left( a b \sigma +a c\beta +b c \gamma -a \kappa +a^2 \delta _1+b^2 \delta _4+c^2 \rho \right) }{a^2 \delta _2 \tau _2},$$
  $$\mathbb {C}_2= \frac{3 \sqrt{\tau _4} \left( a b \sigma +a c\beta +b c \gamma -a \kappa +a^2 \delta _1+b^2 \delta _4+c^2 \rho \right) }{a^2 \delta _2 \tau _2},\ \delta _3= -\frac{\left( a b \sigma +a c\beta +b c \gamma -a \kappa +a^2 \delta _1+b^2 \delta _4+c^2 \rho \right) }{a^4 \tau _2}.$$

Using the solution set (1.1), the solutions to Eq. (1) can be expressed as follows:

(**1.1,1**): If $\tau _2> 0,\ \tau _4 < 0$ and $a^2 \delta _2\ne 0$, the bright soliton that follows is then produced:
  10 $$\begin{aligned} \Phi _{1.1,1}(x,y,z,t)= & -\frac{3 \left( a b \sigma +a c\beta +b c \gamma -a \kappa +a^2 \delta _1+b^2 \delta _4+c^2 \rho \right) }{2 a^2 \delta _2} \nonumber \\ & \text {sech}^2\left[ \left( a x+b y+c z-\frac{\kappa t^{\alpha }}{\alpha }\right) \sqrt{\tau _2}\right] . \end{aligned}$$
(**1.1,2**): If $\tau _2<0,\ \tau _4> 0$ and $a^2 \delta _2\ne 0$, then the ensuing singular periodic solution is acquired:
  11 $$\begin{aligned} \Phi _{1.1,2}(x,y,z,t)= & -\frac{3 \left( a b \sigma +a c\beta +b c \gamma -a \kappa +a^2 \delta _1+b^2 \delta _4+c^2 \rho \right) }{2 a^2 \delta _2} \nonumber \\ & \text {sec}^2\left[ \left( a x+b y+c z-\frac{\kappa t^{\alpha }}{\alpha }\right) \sqrt{-\tau _2}\right] . \end{aligned}$$
(1.1,3): If $\tau _2 <0,\ \tau _4> 0$ and $a^2 \delta _2\ne 0$ then, the offered solution is:
  12 $$\begin{aligned} \displaystyle \Phi _{1.1,3}(x,y,z,t)= & -\frac{3 \left( a b \sigma +a c\beta +b c \gamma -a \kappa +a^2 \delta _1+b^2 \delta _4+c^2 \rho \right) }{2 a^2 \delta _2} \nonumber \\ & \text {csc}^2\left[ \left( a x+b y+c z-\frac{\kappa t^{\alpha }}{\alpha }\right) \sqrt{-\tau _2}\right] , \end{aligned}$$
  this solution denotes a singular periodic solution.

The solution set (1.2) is used to raise the following, which are solutions to Eq. (1):

(**1.2,1**): If $\tau _2<0,\ \tau _4> 0$, $a^2 \delta _2\ne 0$ and $\text {sin}\left[ \left( a x+b y+c z-\frac{\kappa t^{\alpha }}{\alpha }\right) \sqrt{-\tau _2}\right] \ne -1$, the resulting periodic wave solution is as follows:
  13 $$\begin{aligned} \Phi _{1.2,1}(x,y,z,t)=-\frac{3 \left( a b \sigma +a c\beta +b c \gamma -a \kappa +a^2 \delta _1+b^2 \delta _4+c^2 \rho \right) }{a^2 \delta _2 \left( 1+\text {sin}\left[ \left( a x+b y+c z-\frac{\kappa t^{\alpha }}{\alpha }\right) \sqrt{-\tau _2}\right] \right) }. \end{aligned}$$
(**1.2,2**): If $\tau _2 <0,\ \tau _4> 0$, $a^2 \delta _2\ne 0$ and $\text {cos}\left[ \left( a x+b y+c z-\frac{\kappa t^{\alpha }}{\alpha }\right) \sqrt{-\tau _2}\right] \ne 1$, then the below periodic wave solution is produced:
  14 $$\begin{aligned} \displaystyle \Phi _{1.2,2}(x,y,z,t)=-\frac{3 \left( a b \sigma +a c\beta +b c \gamma -a \kappa +a^2 \delta _1+b^2 \delta _4+c^2 \rho \right) }{2 a^2 \delta _2 \left( 1-\text {cos}\left[ \left( a x+b y+c z-\frac{\kappa t^{\alpha }}{\alpha }\right) \sqrt{-\tau _2}\right] \right) }. \end{aligned}$$

**Case (2):** When $\tau _1 = \tau _3 = \tau _6 =0,\ \tau _0 = \dfrac{\tau _2^2}{4\tau _4}$, the following sets of solutions are obtained:

(**2.1**): $$\mathbb {A}_1=\mathbb {B}_1=\mathbb {B}_2=\mathbb {C}_2=\mathbb {D}_1=\mathbb {D}_2=0,\ \mathbb {A}_0=-\frac{3 a^2 \delta _3 \tau _2}{\delta _2},\ \mathbb {A}_2=-\frac{6 a^2 \delta _3 \tau _4}{\delta _2},$$
  $$\sigma =-\frac{a c\beta +b c \gamma -a \kappa +a^2 \delta _1+b^2 \delta _4+c^2 \rho -2 a^4 \delta _3 \tau _2 }{a b}.$$
(**2.2**): $$\mathbb {A}_1=\mathbb {B}_1=\mathbb {B}_2=\mathbb {C}_2=\mathbb {D}_1=\mathbb {D}_2=0,\ \mathbb {A}_0=-\frac{a^2 \delta _3 \tau _2}{\delta _2},\ \mathbb {A}_2=-\frac{6 a^2 \delta _3 \tau _4}{\delta _2},$$
  $$\sigma =-\frac{a c\beta +b c \gamma -a \kappa +a^2 \delta _1+b^2 \delta _4+c^2 \rho +2 a^4 \delta _3 \tau _2 }{a b}.$$
(**2.3**): $$\mathbb {A}_1=\mathbb {B}_1=\mathbb {C}_2=\mathbb {D}_1=\mathbb {D}_2=0,\ \mathbb {A}_0=-\frac{6 a^2 \delta _3 \tau _2}{\delta _2},\ \mathbb {A}_2=-\frac{6 a^2 \delta _3 \tau _4}{\delta _2},\ \mathbb {B}_2=-\frac{3 a^2 \delta _3 \tau _2^2}{2 \delta _2 \tau _4},$$
  $$\sigma =-\frac{a c\beta +b c \gamma -a \kappa +a^2 \delta _1+b^2 \delta _4+c^2 \rho -8 a^4 \delta _3 \tau _2 }{a b}.$$
(**2.4**): $$\mathbb {A}_1=\mathbb {A}_2=\mathbb {B}_1=\mathbb {C}_2=\mathbb {D}_1=\mathbb {D}_2=0,\ \mathbb {A}_0=-\frac{a^2 \delta _3 \tau _2}{\delta _2},\ \mathbb {B}_2=-\frac{3 a^2 \delta _3 \tau _2^2}{2 \delta _2 \tau _4},$$
  $$\sigma =-\frac{a c\beta +b c \gamma -a \kappa +a^2 \delta _1+b^2 \delta _4+c^2 \rho +2 a^4 \delta _3 \tau _2}{a b}.$$
(**2.5**): $$\mathbb {A}_1=\mathbb {B}_1=\mathbb {C}_2=\mathbb {D}_1=\mathbb {D}_2=0,\ \mathbb {A}_0=\frac{2 a^2 \delta _3 \tau _2}{\delta _2},\ \mathbb {A}_2=-\frac{6 a^2 \delta _3 \tau _4}{\delta _2},\ \mathbb {B}_2=-\frac{3 a^2 \delta _3 \tau _2^2}{2 \delta _2 \tau _4}$$
  $$\sigma =-\frac{a c\beta +b c \gamma -a \kappa +a^2 \delta _1+b^2 \delta _4+c^2 \rho +8 a^4 \delta _3 \tau _2}{a b}.$$

The answers for Eq. (1) can be represented using the solution set (2.1) as follows:

(**2.1,1**): If $\tau _2 < 0,\ \tau _4> 0$ and $\delta _2\ne 0$, the bright soliton solution that follows is raised:
  15 $$\begin{aligned} \Phi _{2.1,1}(x,y,z,t)=-\frac{3 a^2 \delta _3 \tau _2}{\delta _2}\ \text {sech}^2\left[ \left( a x+b y+c z-\frac{\kappa t^{\alpha }}{\alpha }\right) \sqrt{-\frac{\tau _2}{2}}\right] . \end{aligned}$$
(**2.1,2**): If $\tau _2> 0,\ \tau _4> 0$ and $\delta _2\ne 0$, then the solution is given as the below singular periodic solution :
  16 $$\begin{aligned} \Phi _{2.1,2}(x,y,z,t)=-\frac{3 a^2 \delta _3 \tau _2}{\delta _2}\ \text {sec}^2\left[ \left( a x+b y+c z-\frac{\kappa t^{\alpha }}{\alpha }\right) \sqrt{\frac{\tau _2}{2}}\right] . \end{aligned}$$

The solution set (2.2) can express the solutions of Eq. (1) as:

(**2.2,1**): If $\tau _2 < 0,\ \tau _4> 0$ and $\delta _2\ne 0$, then a dark soliton solution is raised in the following way:
  17 $$\begin{aligned} \displaystyle \Phi _{2.2,1}(x,y,z,t)=\frac{a^2 \delta _3 \tau _2}{\delta _2} \left( -1+3 \tanh ^2\left[ \left( a x+b y+c z-\frac{\kappa t^{\alpha }}{\alpha }\right) \sqrt{-\frac{\tau _2}{2}} \right] \right) . \end{aligned}$$
(**2.2,2**): If $\tau _2> 0,\ \tau _4> 0$ and $\delta _2\ne 0$, then the solution is:
  18 $$\begin{aligned} \displaystyle \Phi _{2.2,2}(x,y,z,t)=-\frac{a^2 \delta _3 \tau _2}{\delta _2} \left( 1+3 \tan ^2\left[ \left( a x+b y+c z-\frac{\kappa t^{\alpha }}{\alpha }\right) \sqrt{\frac{\tau _2}{2}} \right] \right) , \end{aligned}$$
  this solution is an example of a singular periodic solution.

The following solutions to Eq. (1) can be found by applying the solution set (2.3).

(**2.3,1**): If $\tau _2 < 0,\ \tau _4> 0$ and $\delta _2\ne 0$, the below singular soliton solution is produced:
  19 $$\begin{aligned} \displaystyle \Phi _{2.3,1}(x,y,z,t)=\frac{12 a^2 \delta _3 \tau _2}{\delta _2} {{\,\textrm{csch}\,}}^2\left[ \left( a x+b y+c z-\frac{\kappa t^{\alpha }}{\alpha }\right) \sqrt{-2\tau _2} \right] . \end{aligned}$$
(**2.3,2**): If $\tau _2> 0,\ \tau _4> 0$ and $\delta _2\ne 0$, the following singular periodic solution is found:
  20 $$\begin{aligned} \displaystyle \Phi _{2.3,2}(x,y,z,t)=-\frac{12 a^2 \delta _3 \tau _2}{\delta _2} \csc ^2\left[ \left( a x+b y+c z-\frac{\kappa t^{\alpha }}{\alpha }\right) \sqrt{2\tau _2} \right] . \end{aligned}$$

Using solution set (2.4), the solutions to equation (1) appear as:

(**2.4,1**): If $\tau _2 < 0,\ \tau _4> 0$ and $\delta _2\ne 0$, we obtained a singular soliton solution as below:
  21 $$\begin{aligned} \displaystyle \Phi _{2.4,1}(x,y,z,t)=\frac{a^2 \delta _3 \tau _2}{\delta _2}\left( -1+3 \coth ^2\left[ \left( a x+b y+c z-\frac{\kappa t^{\alpha }}{\alpha }\right) \sqrt{-\frac{\tau _2}{2}} \right] \right) . \end{aligned}$$
(**2.4,2**): If $\tau _2> 0,\ \tau _4> 0$ and $\delta _2\ne 0$, It yields the singular periodic solution as follows:
  22 $$\begin{aligned} \displaystyle \Phi _{2.4,2}(x,y,z,t)=-\frac{a^2 \delta _3 \tau _2}{\delta _2}\left( 1+3 \cot ^2\left[ \left( a x+b y+c z-\frac{\kappa t^{\alpha }}{\alpha }\right) \sqrt{\frac{\tau _2}{2}} \right] \right) . \end{aligned}$$

Eq. (1) solutions can be expressed using the set of solutions (2.5) as follows:

(**2.5,1**): If $\tau _2 < 0,\ \tau _4> 0$ and $\delta _2\ne 0$, then, the solution is:
  23 $$\begin{aligned} \displaystyle \Phi _{2.5,1}(x,y,z,t)=\frac{4 a^2 \delta _3 \tau _2}{\delta _2}\left( -1+3 \coth ^2\left[ \left( a x+b y+c z-\frac{\kappa t^{\alpha }}{\alpha }\right) \sqrt{-2 \tau _2} \right] \right) , \end{aligned}$$
  where a singular soliton solution is represented by this solution.
(**2.5,2**): If $\tau _2> 0,\ \tau _4> 0$ and $\delta _2\ne 0$, the singular periodic solution shown below is produced:
  24 $$\begin{aligned} \displaystyle \Phi _{2.5,2}(x,y,z,t)=\frac{4 a^2 \delta _3 \tau _2}{\delta _2}\left( 2-3 \csc ^2\left[ \left( a x+b y+c z-\frac{\kappa t^{\alpha }}{\alpha }\right) \sqrt{2 \tau _2} \right] \right) . \end{aligned}$$

**Case (3):** When $\tau _3=\tau _4 = \tau _6 = 0$, the discovered set of solutions is as follows:

$$\begin{aligned} \displaystyle \mathbb {A}_0= & \mathbb {A}_1=\mathbb {A}_2=\mathbb {C}_2=\mathbb {D}_1=0,\ \mathbb {B}_1=\frac{3 \tau _1 \left( a b \sigma +a c\beta +b c \gamma -a \kappa +a^2 \delta _1+b^2 \delta _4+c^2 \rho \right) }{2 a^2 \delta _2 \tau _2}, \\ \mathbb {B}_2= & \frac{3 \tau _0 \left( a b \sigma +a c\beta +b c \gamma -a \kappa +a^2 \delta _1+b^2 \delta _4+c^2 \rho \right) }{a^2 \delta _2 \tau _2},\\ \mathbb {D}_2= & \frac{3 \sqrt{\tau _0} \left( a b \sigma +a c\beta +b c \gamma -a \kappa +a^2 \delta _1+b^2 \delta _4+c^2 \rho \right) }{a^2 \delta _2 \tau _2},\\ \delta _3= & -\frac{a b \sigma +a c\beta +b c \gamma -a \kappa +a^2 \delta _1+b^2 \delta _4+c^2 \rho }{a^4 \tau _2}, \end{aligned}$$

then, Eq. (1) appears with the following solutions:

(**3.1**): If $\tau _0=0,\ \tau _2> 0,$ $a^2 \delta _2\ne 0$ and $\sinh \left[ \left( a x+b y+c z-\frac{\kappa t^{\alpha }}{\alpha }\right) \sqrt{4 \tau _2}\right] \ne 1$, the following hyperbolic solution is produced:
  25 $$\begin{aligned} \displaystyle \Phi _{3.1}(x,y,z,t)=\frac{3 \left( a b \sigma +a c\beta +b c \gamma -a \kappa +a^2 \delta _1+b^2 \delta _4+c^2 \rho \right) }{a^2 \delta _2 \left( \sinh \left[ \left( a x+b y+c z-\frac{\kappa t^{\alpha }}{\alpha }\right) \sqrt{4 \tau _2}\right] -1\right) }. \end{aligned}$$
(**3.2**): If $\tau _0=0,\ \tau _2 < 0,$ $a^2 \delta _2\ne 0$ and $\sin \left[ \left( a x+b y+c z-\frac{\kappa t^{\alpha }}{\alpha }\right) \sqrt{-4 \tau _2}\right] \ne 1$, the periodic wave solution is then raised:
  26 $$\begin{aligned} \displaystyle \Phi _{3.2}(x,y,z,t)= & \frac{3 \left( a b \sigma +a c\beta +b c \gamma -a \kappa +a^2 \delta _1+b^2 \delta _4+c^2 \rho \right) }{a^2 \delta _2 \left( \sin \left[ \left( a x+b y+c z-\frac{\kappa t^{\alpha }}{\alpha }\right) \sqrt{-4 \tau _2}\right] -1\right) }. \end{aligned}$$
(**3.3**): If $\tau _0>0,\ \tau _2> 0,\ \tau _1=0$ and $a^2 \delta _2 \sinh \left[ \left( a x+b y+c z-\frac{\kappa t^{\alpha }}{\alpha }\right) \sqrt{\tau _2} \right] \ne 0$, then, the following hyperbolic solution is found:
  27 $$\begin{aligned} \displaystyle \Phi _{3.3}(x,y,z,t)= & \frac{3 \left( a b \sigma +a c\beta +b c \gamma -a \kappa +a^2 \delta _1+b^2 \delta _4+c^2 \rho \right) }{a^2 \delta _2} \nonumber \\ & \left( \frac{1+\cosh \left[ \left( a x+b y+c z-\frac{\kappa t^{\alpha }}{\alpha }\right) \sqrt{\tau _2}\right] }{\sinh ^2\left[ \left( a x+b y+c z-\frac{\kappa t^{\alpha }}{\alpha }\right) \sqrt{\tau _2}\right] }\right) . \end{aligned}$$
(**3.4**): If $\tau _0>0,\ \tau _2 < 0,\ \tau _1=0$ and $a^2 \delta _2 \sin \left[ \left( a x+b y+c z-\frac{\kappa t^{\alpha }}{\alpha }\right) \sqrt{\tau _2} \right] \ne 0$, the periodic wave solution type is derived as follows:
  28 $$\begin{aligned} \displaystyle \Phi _{3.4}(x,y,z,t)= & -\frac{3\left( a b \sigma +a c\beta +b c \gamma -a \kappa +a^2 \delta _1+b^2 \delta _4+c^2 \rho \right) }{a^2 \delta _2} \nonumber \\ & \left( \frac{1+\cos \left[ \left( a x+b y+c z-\frac{\kappa t^{\alpha }}{\alpha }\right) \sqrt{-\tau _2}\right] }{\sin ^2\left[ \left( a x+b y+c z-\frac{\kappa t^{\alpha }}{\alpha }\right) \sqrt{-\tau _2}\right] }\right) . \end{aligned}$$
(**3.5**): If $\tau _2>0,\ \tau _0=\frac{\tau _1^2}{4 \tau _2}$ and $a^2 \delta _2 \left( \tau _1-2 \tau _2 e^{\left( a x+b y+c z-\frac{\kappa t^{\alpha }}{\alpha }\right) \sqrt{\tau _2}}\right) \ne 0$, then the solution is:
  29 $$\begin{aligned} \displaystyle \Phi _{3.5}(x,y,z,t)= & \frac{12 \tau _1 \tau _2 \left( a b \sigma +a c\beta +b c \gamma -a \kappa +a^2 \delta _1+b^2 \delta _4+c^2 \rho \right) }{a^2 \delta _2} \nonumber \\ & \frac{e^{\left( a x+b y+c z-\frac{\kappa t^{\alpha }}{\alpha }\right) \sqrt{\tau _2} }}{\left( \tau _1-2 \tau _2 e^{\left( a x+b y+c z-\frac{\kappa t^{\alpha }}{\alpha }\right) \sqrt{\tau _2}}\right) {}^2}. \end{aligned}$$
  this solution represents an exponential wave solution.

**Case (4):** When $\tau _0 = \tau _1 = \tau _2 = \tau _6 =0$, then following set of solutions is found:

$$\displaystyle \mathbb {A}_0=\mathbb {B}_1=\mathbb {B}_2=\mathbb {D}_1=\mathbb {D}_2=0,\ \mathbb {A}_1=-\frac{3 a^2 \delta _3 \tau _3}{2 \delta _2},\ \mathbb {A}_2=-\frac{3 a^2 \delta _3 \tau _4}{\delta _2},\ \mathbb {C}_2=\frac{3 a^2 \delta _3 \sqrt{\tau _4}}{\delta _2},$$

$$\sigma =-\frac{a c\beta +b c \gamma -a \kappa +a^2 \delta _1+b^2 \delta _4+c^2 \rho }{a b}.$$

The solution to Equation (1) is:

30 $$\begin{aligned} \displaystyle \Phi _{4}(x,y,z,t)=-\frac{6 a^2 \delta _3 \tau _3^2}{\delta _2 \left( \tau _3 \left( a x+b y+c z-\frac{\kappa t^{\alpha }}{\alpha }\right) -2 \sqrt{\tau _4}\right) {}^2} , \end{aligned}$$

which represents a rational wave solution, such that $\delta _2 \left( \tau _3 \left( a x+b y+c z-\frac{\kappa t^{\alpha }}{\alpha }\right) -2 \sqrt{\tau _4}\right) \ne 0$.

**Case (5):** When $\tau _0 = \tau _1 = \tau _6 = 0$, then the following solution sets are discovered:

(**5.1**): $$\mathbb {A}_0=\mathbb {B}_1=\mathbb {B}_2=\mathbb {D}_1=\mathbb {D}_2=0,\ \mathbb {A}_1=-\frac{3 \left( a b \sigma +a c\beta +b c \gamma -a \kappa +a^2 \delta _1+b^2 \delta _4+c^2 \rho \right) \sqrt{\tau _4}}{a^2 \delta _2 \sqrt{\tau _2}},$$
  $$\mathbb {A}_2=\frac{3 \tau _4 \left( a b \sigma +a c\beta +b c \gamma -a \kappa +a^2 \delta _1+b^2 \delta _4+c^2 \rho \right) }{a^2 \delta _2 \tau _2},\ \mathbb {C}_2=-\frac{3 \left( a b \sigma +a c\beta +b c \gamma -a \kappa +a^2 \delta _1+b^2 \delta _4+c^2 \rho \right) \sqrt{\tau _4} }{a^2 \delta _2 \tau _2},$$
  $$\delta _3= -\frac{a b \sigma +a c\beta +b c \gamma -a \kappa +a^2 \delta _1+b^2 \delta _4+c^2 \rho }{4 a^4 \tau _2},\ \tau _3= -2 \sqrt{\tau _2 \tau _4}.$$
(**5.2**): $$\mathbb {B}_1=\mathbb {B}_2=\mathbb {C}_2=\mathbb {D}_1=\mathbb {D}_2=0,\ \mathbb {A}_0=-\frac{a b \sigma +a c\beta +b c \gamma -a \kappa +a^2 \delta _1+b^2 \delta _4+c^2 \rho }{a^2 \delta _2},$$
  $$\mathbb {A}_1=-\frac{6 \left( a b \sigma +a c\beta +b c \gamma -a \kappa +a^2 \delta _1+b^2 \delta _4+c^2 \rho \right) \sqrt{\tau _4} }{a^2 \delta _2 \sqrt{\tau _2}},\ \mathbb {A}_2=\frac{6 \tau _4 \left( a b \sigma +a c\beta +b c \gamma -a \kappa +a^2 \delta _1+b^2 \delta _4+c^2 \rho \right) }{a^2 \delta _2 \tau _2},$$
  $$\delta _3= -\frac{a b \sigma +a c\beta +b c \gamma -a \kappa +a^2 \delta _1+b^2 \delta _4+c^2 \rho }{a^4 \tau _2},\ \tau _3= 2 \sqrt{\tau _2 \tau _4}.$$
(**5.3**): $$\mathbb {A}_0=\mathbb {A}_1=\mathbb {B}_1=\mathbb {B}_2=\mathbb {C}_2=\mathbb {D}_1=\mathbb {D}_2=\tau _3=0,\ \mathbb {A}_2=\frac{3 \tau _4 \left( a b \sigma +a c\beta +b c \gamma -a \kappa +a^2 \delta _1+b^2 \delta _4+c^2 \rho \right) }{2a^2 \delta _2 \tau _2},$$
  $$\delta _3= -\frac{a b \sigma +a c\beta +b c \gamma -a \kappa +a^2 \delta _1+b^2 \delta _4+c^2 \rho }{4a^4 \tau _2}.$$

The solution set (5.1) can be used for Eq. (1) to express its solutions as follows:

(**5.1**): If $\tau _2> 0$, $\tau _4> 0$ and $a \delta _2\ne 0$, consequently, the following are the solutions:
  31 $$\begin{aligned} \displaystyle \Phi _{5.1,1}(x,y,z,t)=-\frac{3 \left( a b \sigma +a c\beta +b c \gamma -a \kappa +a^2 \delta _1+b^2 \delta _4+c^2 \rho \right) }{2a^2 \delta _2}\ \text {sech}^2\left[ \left( a x+b y+c z-\frac{\kappa t^{\alpha }}{\alpha }\right) \sqrt{\frac{\tau _2}{4}}\right] , \end{aligned}$$
  where this solution represents a bright soliton solution,
  or
  32 $$\begin{aligned} \displaystyle \Phi _{5.1,2}(x,y,z,t)=\frac{3 \left( a b \sigma +a c\beta +b c \gamma -a \kappa +a^2 \delta _1+b^2 \delta _4+c^2 \rho \right) }{2a^2 \delta _2}\ \text {csch}^2\left[ \left( a x+b y+c z-\frac{\kappa t^{\alpha }}{\alpha }\right) \sqrt{\frac{\tau _2}{4}}\right] , \end{aligned}$$
  where the solution acts as a singular soliton solution.
(**5.2,1**): If $\tau _2> 0$, $\tau _4> 0$ and $a \delta _2\ne 0$, a dark soliton solution is then obtained as:
  33 $$\begin{aligned} \displaystyle \Phi _{5.2,1}(x,y,z,t)= & -\frac{ \left( a b \sigma +a c\beta +b c \gamma -a \kappa +a^2 \delta _1+b^2 \delta _4+c^2 \rho \right) }{2a^2 \delta _2} \nonumber \\ & \left( -1+ 3\ \text {tanh}^2\left[ \left( a x+b y+c z-\frac{\kappa t^{\alpha }}{\alpha }\right) \sqrt{\frac{\tau _2}{4}}\right] \right) . \end{aligned}$$
(**5.2,2**): If $\tau _2> 0$, $\tau _4> 0$ and $a \delta _2\ne 0$, the below singular soliton solution is obtained:
  34 $$\begin{aligned} \displaystyle \Phi _{5.2,2}(x,y,z,t)= & \frac{3 \left( a b \sigma +a c\beta +b c \gamma -a \kappa +a^2 \delta _1+b^2 \delta _4+c^2 \rho \right) }{2a^2 \delta _2} \nonumber \\ & \left( 1- 3\ \text {coth}^2\left[ \left( a x+b y+c z-\frac{\kappa t^{\alpha }}{\alpha }\right) \sqrt{\frac{\tau _2}{4}}\right] \right) . \end{aligned}$$
(**5.3,1**): If $\tau _2> 0$, $\tau _4> 0$, $\tau _3^2\ne 4 \tau _2 \tau _4$ and $a \delta _2\ne 0$, the singular soliton solution that follows is produced:
  35 $$\begin{aligned} \displaystyle \Phi _{5.3,1}(x,y,z,t)= & \frac{3 \left( a b \sigma +a c\beta +b c \gamma -a \kappa +a^2 \delta _1+b^2 \delta _4+c^2 \rho \right) }{2a^2 \delta _2} \nonumber \\ & \text {csch}^2\left[ \left( a x+b y+c z-\frac{\kappa t^{\alpha }}{\alpha }\right) \sqrt{\tau _2}\right] . \end{aligned}$$
(**5.3,2**): If $\tau _2 < 0$, $\tau _4> 0$, $\tau _3^2\ne 4 \tau _2 \tau _4$ and $a \delta _2\ne 0$, a singular periodic solution is produced as:
  36 $$\begin{aligned} \displaystyle \Phi _{5.3,2}(x,y,z,t)= & -\frac{3 \left( a b \sigma +a c\beta +b c \gamma -a \kappa +a^2 \delta _1+b^2 \delta _4+c^2 \rho \right) }{2a^2 \delta _2}\nonumber \\ & \text {csc}^2\left[ \left( a x+b y+c z-\frac{\kappa t^{\alpha }}{\alpha }\right) \sqrt{-\tau _2}\right] . \end{aligned}$$

**Case (6):** When $\tau _2 = \tau _4 =\tau _6 = 0$, we discovered the following set of solutions:

$$\begin{aligned} \mathbb {A}_2= & \mathbb {B}_1=\mathbb {B}_2=\mathbb {C}_2=\mathbb {D}_1=\mathbb {D}_2=0,\ \mathbb {A}_0=-\frac{a b \sigma +a c\beta +b c \gamma -a \kappa +a^2 \delta _1+b^2 \delta _4+c^2 \rho }{2 a^2 \delta _2}, \\ \mathbb {A}_1= & \frac{\left( a b \sigma +a c\beta +b c \gamma -a \kappa +a^2 \delta _1+b^2 \delta _4+c^2 \rho \right) {}^2}{2 a^6 \delta _2 \delta _3 \tau _1},\\ \tau _3= & -\frac{\left( a b \sigma +a c\beta +b c \gamma -a \kappa +a^2 \delta _1+b^2 \delta _4+c^2 \rho \right) {}^2}{3 a^8 \delta _3^2 \tau _1}. \end{aligned}$$

Then, the solution is:

37 $$\begin{aligned} \begin{aligned} \Phi _{6}(x,y,z) =&\ \frac{\left( a b \sigma + a c \beta + b c \gamma - a \kappa + a^2 \delta _1 + b^2 \delta _4 + c^2 \rho \right) }{2 a^6 \delta _2} \\ &\left( -a^4 + \frac{a b \sigma + a c \beta + b c \gamma - a \kappa + a^2 \delta _1 + b^2 \delta _4 + c^2 \rho }{\delta _3 \tau _1} \right. \\&\ \left. \wp \left[ \left( a x + b y + c z - \frac{\kappa t^{\alpha }}{\alpha } \right) \sqrt{ -\frac{\left( a b \sigma + a c \beta + b c \gamma - a \kappa + a^2 \delta _1 + b^2 \delta _4 + c^2 \rho \right) ^2}{12 a^8 \delta _3^2 \tau _1} }, \right. \right. \\&\ \left. \left. \frac{12 a^8 \delta _3^2 \tau _1^2}{\left( a b \sigma + a c \beta + b c \gamma - a \kappa + a^2 \delta _1 + b^2 \delta _4 + c^2 \rho \right) ^2}, \right. \right. \\&\ \left. \left. \frac{12 a^8 \delta _3^2 \tau _0 \tau _1}{\left( a b \sigma + a c \beta + b c \gamma - a \kappa + a^2 \delta _1 + b^2 \delta _4 + c^2 \rho \right) ^2} \right] \right) , \end{aligned} \end{aligned}$$

this solution represents a doubly periodic Weierstrass elliptic solution in the conditions of

$$\tau _1<0$$, $$\tau _3>0$$ and $$a \delta _2 \delta _3\ne 0$$.

**Case (7):** When $\tau _1 = \tau _3 = 0$, the below set of solutions is generated:

$$\mathbb {A}_1=\mathbb {A}_2=\mathbb {B}_1=\mathbb {C}_2=\mathbb {D}_1=\mathbb {D}_2=0,\ \mathbb {A}_0=-\frac{a b \sigma +a c \beta +b c \gamma -a \kappa +a^2 \delta _1+ +b^2 \delta _4 +c^2\rho +4 a^4 \delta _3 \tau _2 }{2 a^2 \delta _2},$$

$$\mathbb {B}_2=\frac{\left( a b \sigma +a c \beta +b c \gamma -a \kappa +a^2 \delta _1+ +b^2 \delta _4 +c^2\rho -4 a^4 \delta _3 \tau _2 \right) \left( a b \sigma +a c \beta +b c \gamma -a \kappa +a^2 \delta _1+ +b^2 \delta _4 +c^2\rho +4 a^4 \delta _3 \tau _2 \right) }{8 a^6 \delta _2 \delta _3 \tau _4},$$

$$\tau _0=-\frac{\left( a b \sigma +a c \beta +b c \gamma -a \kappa +a^2 \delta _1+ +b^2 \delta _4 +c^2\rho -4 a^4 \delta _3 \tau _2 \right) \left( a b \sigma +a c \beta +b c \gamma -a \kappa +a^2 \delta _1+ +b^2 \delta _4 +c^2\rho +4 a^4 \delta _3 \tau _2 \right) }{48 a^8 \delta _3^2 \tau _4}.$$

The following solutions for Eq. (1) can be derived using the set of solutions above:

(**7.1**): If $\tau _2>0$, $\tau _4^2>4 \tau _2 \tau _6$, and $a \delta _2 \delta _3 \tau _2 \tau _4\ne 0$, a hyperbolic solution or a periodic wave solution is located as follows, respectively:
  38 $$\begin{aligned} & \begin{aligned} \Phi _{7.1} (x,y,z)=&\ \frac{\left( a b \sigma + a c \beta + b c \gamma - a \kappa + a^2 \delta _1 + b^2 \delta _4 + c^2 \rho + 4 a^4 \delta _3 \tau _2 \right) }{16 a^6 \delta _2} \\&\ \times \left( -8 a^4 - \frac{a b \sigma + a c \beta + b c \gamma - a \kappa + a^2 \delta _1 + b^2 \delta _4 + c^2 \rho - 4 a^4 \delta _3 \tau _2}{\delta _3 \tau _2 \tau _4} \right. \\&\ \left. \times \left( \tau _4 - \cosh \left[ \left( a x + b y + c z - \frac{\kappa t^{\alpha }}{\alpha } \right) \sqrt{4 \tau _2} \right] \sqrt{\tau _4^2 - 4 \tau _2 \tau _6} \right) \right) \end{aligned} \end{aligned}$$
  39 $$\begin{aligned} & \begin{aligned} \Phi _{7.2} (x,y,z)=&\ \frac{\left( a b \sigma + a c \beta + b c \gamma - a \kappa + a^2 \delta _1 + b^2 \delta _4 + c^2 \rho + 4 a^4 \delta _3 \tau _2 \right) }{16 a^6 \delta _2} \\&\ \times \left( -8 a^4 - \frac{a b \sigma + a c \beta + b c \gamma - a \kappa + a^2 \delta _1 + b^2 \delta _4 + c^2 \rho - 4 a^4 \delta _3 \tau _2}{\delta _3 \tau _2 \tau _4} \right. \\&\ \left. \times \left( \tau _4 - \cos \left[ \left( a x + b y + c z - \frac{\kappa t^{\alpha }}{\alpha } \right) \sqrt{4 \tau _2} \right] \sqrt{\tau _4^2 - 4 \tau _2 \tau _6} \right) \right) \end{aligned} \end{aligned}$$

**Case (8):** When $\tau _1 = \tau _3 =\tau _6 = 0$, then a set of solutions is produced as follows:

$$\begin{aligned} & \mathbb {A}_1=\mathbb {A}_2=\mathbb {B}_1=\mathbb {C}_2=\mathbb {D}_1=\mathbb {D}_2= 0,\ \mathbb {A}_0= -\frac{2}{\delta _2}\left( a^2 \delta _3 \tau _2+\sqrt{a^4 \delta _3^2 \left( \tau _2^2-3 \tau _0 \tau _4\right) }\right) ,\ \mathbb {B}_2= -\frac{6 a^2 \delta _3 \tau _0}{\delta _2}, \\ & \rho = -\frac{a (b \sigma +\beta c-\kappa )+b c \gamma +a^2 \left( \delta _1-4 \sqrt{a^4 \delta _3^2 \left( \tau _2^2-3 \tau _0 \tau _4\right) }\right) +b^2 \delta _4}{c^2}. \end{aligned}$$

Through this previous set of solutions, some solutions of Eq. (1) were obtained as:

(**8.1**): If $\tau _0=1,\ \tau _2=-m^2-1,\ \tau _4=m^2, \ \delta _2\ne 0$ and $0\le m\le 1$, then solutions as Jacobi elliptic function are obtained as follows:
  40 $$\begin{aligned} \Phi _{8.1,1}(x,y,z,t)=\frac{2 a^2 \delta _3}{\delta _2}\left[ 1+m^2-\sqrt{m^4-m^2+1}-3\ \text {ns}\left( a x+b y+c z-\frac{\kappa t^{\alpha }}{\alpha }\right) ^2\right] , \end{aligned}$$
  or
  41 $$\begin{aligned} \Phi _{8.1,2}(x,y,z,t)=\frac{2 a^2 \delta _3}{\delta _2}\left[ 1+m^2-\sqrt{m^4-m^2+1}-3\ \text {dc}\left( a x+b y+c z-\frac{\kappa t^{\alpha }}{\alpha }\right) ^2\right] . \end{aligned}$$
  In the special case, Eq. (40) and Eq. (41) yield the singular periodic solutions listed below when $m = 0$:
  42 $$\begin{aligned} \Phi _{8.1,3}(x,y,z,t)=-\frac{6 a^2 \delta _3}{\delta _2} \csc ^2\left[ a x+b y+c z-\frac{\kappa t^{\alpha }}{\alpha }\right] , \end{aligned}$$
  or
  43 $$\begin{aligned} \Phi _{8.1,4}(x,y,z,t)=-\frac{6 a^2 \delta _3}{\delta _2}\ \sec ^2\left[ a x+b y+c z-\frac{\kappa t^{\alpha }}{\alpha }\right] . \end{aligned}$$
  However, Eq. (40) yields the singular soliton as follows for $m = 1$:
  44 $$\begin{aligned} \Phi _{8.1,5}(x,y,z,t)=-\frac{2 a^2 \delta _3}{\delta _2} \left( 2+3\ {{\,\textrm{csch}\,}}^2\left[ a x+b y+c z-\frac{\kappa t^{\alpha }}{\alpha }\right] \right) . \end{aligned}$$
(**8.2**): If $\tau _0=m^2-1,\ \tau _2=2-m^2,\ \tau _4=-1, \ \delta _2\ne 0$ and $0< m< 1$, the following JEF solution is obtained:
  45 $$\begin{aligned} \Phi _{8.2}(x,y,z,t)=\frac{2 a^2 \delta _3}{\delta _2}\left[ m^2 - 2 -\sqrt{m^4-m^2+1}+3 \left( 1-m^2\right) \text {nd}\left( a x+b y+c z-\frac{\kappa t^{\alpha }}{\alpha }\right) ^2\right] . \end{aligned}$$
(**8.3**): If $\tau _0=-m^2,\ \tau _2=2 m^2-1,\ \tau _4=1-m^2, \ \delta _2\ne 0$ and $0< m\le 1$, the JEF solution is presented as follows:
  46 $$\begin{aligned} \Phi _{8.3,1}(x,y,z,t)=\frac{2 a^2 \delta _3}{\delta _2}\left[ \left( 1-2 m^2\right) -\sqrt{m^4-m^2+1}+3m^2\ \text {cn}\left( a x+b y+c z-\frac{\kappa t^{\alpha }}{\alpha }\right) ^2\right] . \end{aligned}$$
  The following bright soliton is obtained as a special case when $m = 1$:
  47 $$\begin{aligned} \Phi _{8.3,2}(x,y,z,t)=-\frac{6 a^2 \delta _3}{\delta _2}\left( 2-3\ {{\,\textrm{sech}\,}}^2\left[ a x+b y+c z-\frac{\kappa t^{\alpha }}{\alpha }\right] \right) . \end{aligned}$$
(**8.4**): If $\tau _0=-1,\ \tau _2=2-m^2,\ \tau _4=m^2-1, \ \delta _2\ne 0$ and $0< m\le 1$, JEF solution is achieved as:
  48 $$\begin{aligned} \Phi _{8.4,1}(x,y,z,t)=\frac{2 a^2 \delta _3}{\delta _2}\left[ \left( m^2-2\right) -\sqrt{m^4-m^2+1}+3 m^2\ \text {dn}\left( a x+b y+c z-\frac{\kappa t^{\alpha }}{\alpha }\right) ^2\right] . \end{aligned}$$
  The following bright soliton is obtained in a special case when $m = 1$:
  49 $$\begin{aligned} \Phi _{8.4,2}(x,y,z,t)=-\frac{6 a^2 \delta _3}{\delta _2}\left( 2-3\ {{\,\textrm{sech}\,}}^2\left[ a x+b y+c z-\frac{\kappa t^{\alpha }}{\alpha }\right] \right) . \end{aligned}$$
(**8.5**): If $\tau _0=\frac{1}{4},\ \tau _2=\frac{1}{2} (m^2 - 2),\ \tau _4=\frac{m^4}{4}, \ \delta _2\ne 0$ and $0\le m< 1$, a solution of a JEF is as follows:
  50 $$\begin{aligned} \Phi _{8.5,1}(x,y,z,t)= & -\frac{a^2 \delta _3}{2 \delta _2}\left[ \left( 2m^2-4\right) +\frac{3 \left( \text {dn}\left( a x+b y+c z-\frac{\kappa t^{\alpha }}{\alpha }\right) ^2 +\sqrt{1-m^2}\right) ^2}{\text {cn}\left( a x+b y+c z-\frac{\kappa t^{\alpha }}{\alpha }\right) ^2} \right. \nonumber \\ & \left. +\sqrt{m^4-16 m^2+16}\right] . \end{aligned}$$
  In the particular instance where $m = 0$, a singular periodic solution that follows is produced:
  51 $$\begin{aligned} \Phi _{8.5,2}(x,y,z,t)=-\frac{6 a^2 \delta _3}{\delta _2} \sec ^2\left[ a x+b y+c z-\frac{\kappa t^{\alpha }}{\alpha }\right] . \end{aligned}$$

## Graphical representations of the solutions

**Fig. 1:**
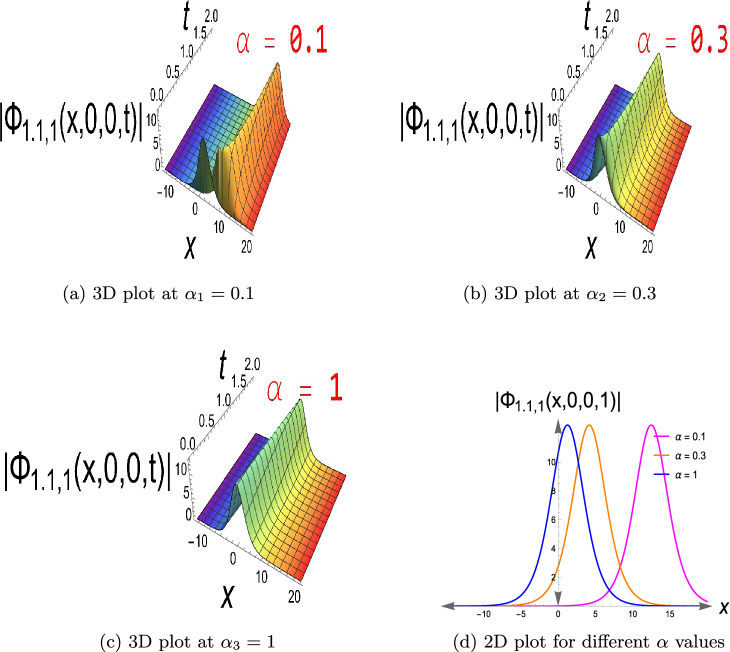
The bright soliton for Eq.(10).

**Fig. 2:**
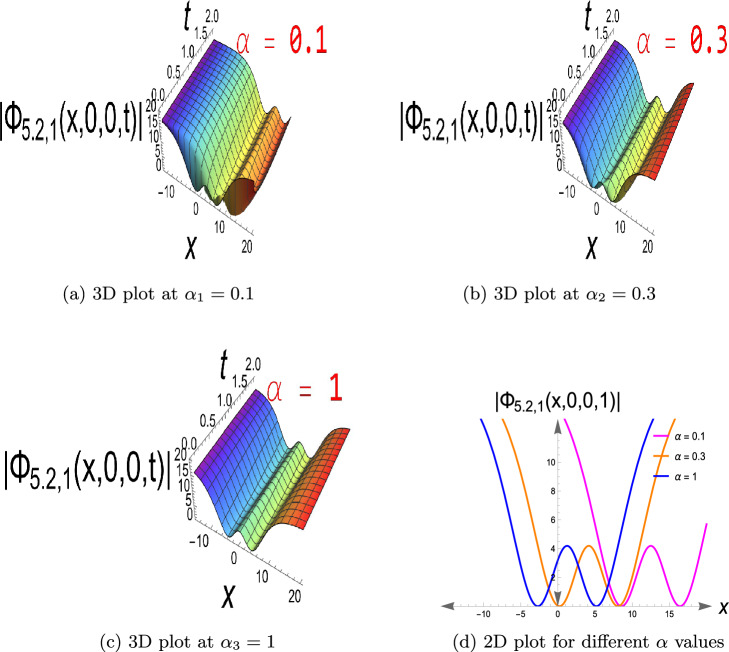
The dark soliton for Eq.(33).

**Fig. 3:**
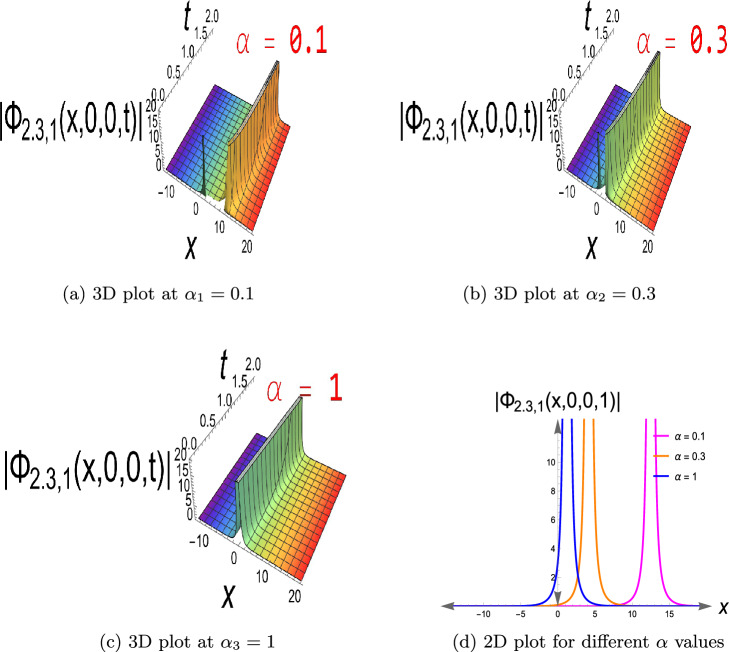
The singular soliton for Eq.(19).

**Fig. 4:**
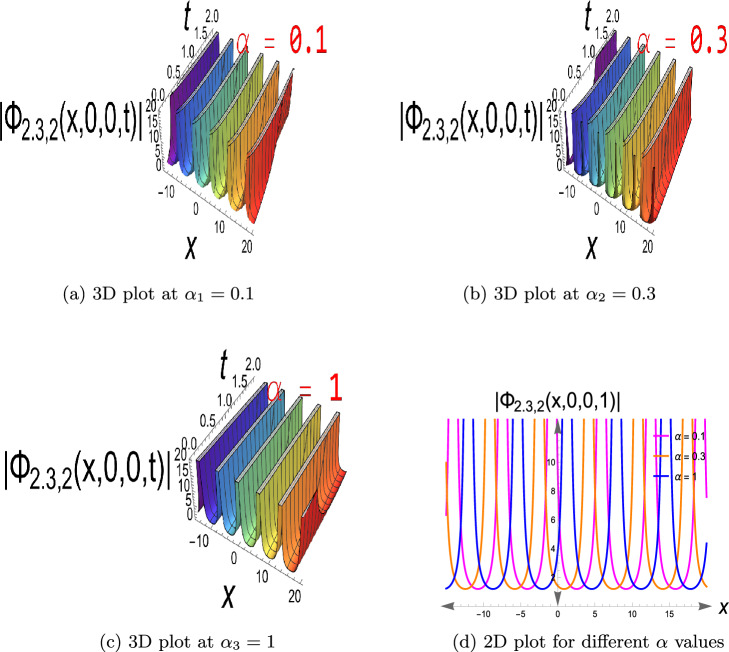
The singular periodic solution for Eq.(20).

Some special solutions are presented in 2D and 3D figures to fully understand their physical structures, which will be displayed. In Fig. 1, a bright soliton of Eq. (10) is displayed when $a=0.4,\ b=0.6,\ c=0.7,\ \beta =1,\ \gamma =0.7,\ \kappa =0.5,$ $\ \rho =0.5,\ \sigma =0.65,\ \delta _1=0.9,$ $\ \delta _2=0.9,\ \delta _4=0.8,\ \tau _2=0.7,\ y=z=0,$ $\ 0< t\le 2,\ -15\le x\le 20$ and $\alpha$ have three different values as, $\alpha _1=0.1,\ \alpha _2=0.3,$ and $\alpha _3=1$ that show the fractional effect in three different values. This type of solution is a localized wave with a single crest in the wave amplitude that vanishes at infinity. Bright solitons arise when nonlinearity and dispersion in a focusing medium (e.g., in optical fibers or shallow water waves) compensate each other. They mimic good pulse propagation and energy localization, which are prerequisites for energy transfer in nonlinear media and optical communications. Figure 2 shows the solution to Eq. (33), a dark soliton, having $a=0.4,\ b=0.6,\ c=0.7,\ \beta =1,\ \gamma =0.7,$ $\ \kappa =0.5,\ \rho =0.5,\ \sigma =0.65,\ \delta _1=0.9,\ \delta _2=0.9,$ $\ \delta _4=0.8,\ \tau _2=0.7,\ y=z=0,\ 0<t<2,$ $\ -15\le x\le 20$ and $\alpha _1=0.1,\ \alpha _2=0.3,\ \alpha _3=1,$ with three distinct values for $\alpha$ in the fractional term. On a continuous wave background, this constitutes a localized depression (intensity hole). It is useful for the simulation of phase-shifted structures, background modulation, and stable transmission channels when intensity reduction is required, e.g., in Bose-Einstein condensates or nonlinear optics with constant background intensity. It usually exists in defocusing nonlinear media. Eq. (19) has a singular soliton, as seen in Fig. 3, choosing the parameters to be $a=0.4,\ b=0.6,\ c=0.7, \kappa =0.5,\delta _2=0.9,\delta _3=0.8,\tau _2=-0.7,\ y=z=0,\ 0< t\le 2$ and $-15\le x\le 20$, also $\alpha$ has values of $\alpha _1=0.1,\ \alpha _2=0.3,$ and $\alpha _3=1$. Singular solitons describe extreme nonlinear effects in which wave amplitude is singular at certain points to account for energy concentration, collapse, or blow-up behavior of physical phenomena in plasma physics, nonlinear optics, and fluid dynamics. Through research on them, critical thresholds are determined at which the system enters unstable or strongly localized states. Furthermore, Fig. 4 depicts Eq.(20), a singular periodic solution, when $a=0.4,\ b=0.6,\ c=0.7,\kappa =0.5,\delta _2=0.9,\delta _3=0.8,\tau _2=0.7,\ y=z=0,\ 0< t\le 2$ and $-15\le x\le 20$, in addition to $\alpha$ with values $\alpha _1=0.1,\ \alpha _2=0.3,\ \alpha _3=1$.Singular periodic solutions provide oscillatory waveforms with periodic behavior and amplitude singularities, which can be utilized to model extreme events such as optical rogue waves, plasma instabilities, or localized energy blasts in nonlinear dispersive media.

## Conclusion

The current study found solitons and additional wave solutions using the modified extended mapping method for a new famous extended (3 + 1)-dimensional equation of Wazwaz, but with the addition of a conformable fractional term, which had not been used before with this new combination. Numerous solutions were extracted, including periodic wave solutions, singular periodic wave solutions, hyperbolic solutions, exponential wave solutions, bright, dark, singular solitons, JEF solutions, Weierstrass elliptic doubly periodic solutions, in addition to rational wave solutions. Some physical attributes have been raised, and some various graphs have been sketched for the obtained solutions. The solutions described in this work are considered novel and have been newly obtained for such a model with its fractional term, in contrast to previous comparable studies. With possible uses in fluid dynamics, optics, plasma physics, and Bose-Einstein condensates, the discovered accurate solutions provide insights into nonlinear wave behavior. These solutions help to better understand and regulate multidimensional nonlinear wave events and act as standards for numerical models.

## Data Availability

The datasets used and/or analysed during the current study available from the corresponding author on reasonable request.
